# Relevance of Cortical and Hippocampal Interneuron Functional Diversity to General Anesthetic Mechanisms: A Narrative Review

**DOI:** 10.3389/fnsyn.2021.812905

**Published:** 2022-01-26

**Authors:** Iris A. Speigel, Hugh C. Hemmings Jr.

**Affiliations:** ^1^Department of Anesthesiology, Weill Cornell Medicine, New York, NY, United States; ^2^Department of Pharmacology, Weill Cornell Medicine, New York, NY, United States

**Keywords:** anesthesia, cerebral cortex, hippocampus, interneurons, isoflurane, ketamine, plasticity

## Abstract

General anesthetics disrupt brain processes involved in consciousness by altering synaptic patterns of excitation and inhibition. In the cerebral cortex and hippocampus, GABAergic inhibition is largely mediated by inhibitory interneurons, a heterogeneous group of specialized neuronal subtypes that form characteristic microcircuits with excitatory neurons. Distinct interneuron subtypes regulate specific excitatory neuron networks during normal behavior, but how these interneuron subtypes are affected by general anesthetics is unclear. This narrative review summarizes current principles of the synaptic architecture of cortical and interneuron subtypes, their contributions to different forms of inhibition, and their roles in distinct neuronal microcircuits. The molecular and cellular targets in these circuits that are sensitive to anesthetics are reviewed in the context of how anesthetics impact interneuron function in a subtype-specific manner. The implications of this functional interneuron diversity for mechanisms of anesthesia are discussed, as are their implications for anesthetic-induced changes in neural plasticity and overall brain function.

## Introduction

The mechanistic understanding of general anesthesia has seen substantial recent progress, but there is a conceptual gap between the molecular pharmacology of anesthetic targets and the network level changes in the anesthetized central nervous system (CNS). General anesthetics alter the balance and dynamics of neural excitation and inhibition at the levels of single receptors, synapses, and circuits. Neural inhibition is largely mediated by GABAergic interneurons in the brain, with biochemically and functionally distinct interneuron subtypes responsible for different forms of synaptic and circuit inhibition. Specialized interneurons are adapted for specific neural circuit roles (Kepecs and Fishell, 2014). Although largely overlooked in studies of anesthetic mechanisms, interneuron diversity has implications for understanding how general anesthetics alter CNS function at the micro-, meso- and macro-circuit levels.

Interneurons release the major inhibitory neurotransmitter gamma-aminobutyric acid (GABA) to stabilize the membrane potential away from the action potential threshold, either by hyperpolarization or shunting inhibition, effectively controlling neurotransmission in target cells. The interneuron function has classically described “local” effects within the same anatomical region as the cell body and dendrites. However, recent findings demonstrate small populations of cortical and hippocampal GABAergic neurons with long-range projections (Jinno, 2009; Melzer and Monyer, 2020). Interneuron cellular and synaptic physiology vary due to differential gene and protein expression, including the expression of several established anesthetic targets, raising the possibility of interneuron subtype-specific and microcircuit-specific roles in determining overall network sensitivities to anesthetic agents and adjuvants. Interneurons have distinct roles in controlling excitatory neuron activity and shaping oscillatory dynamics, and therefore must be considered in explaining anesthetic effects on brain rhythms recorded intraoperatively under anesthesia. Moreover, experimental data suggest that anesthetics differentially modulate interneuron function, as outlined below. As interneuron plasticity has been associated with both positive and negative cognitive outcomes in various animal models (Lewis et al., 2012; Palop and Mucke, 2016; Luscher et al., 2020), these effects have implications for both deleterious and beneficial long-term effects.

Here we review cortical and hippocampal interneuron diversity in the context of the neurophysiological mechanisms and consequences of general anesthesia. We describe the architecture for interneuron specialization, including their heterogeneous expression of known molecular targets for various anesthetics, and discuss evidence of interneuron subtype-specific anesthetic sensitivities determined by differential expression of these anesthetic targets. We also discuss evidence that changes in interneuron function contribute to the persistent effects of anesthesia, and summarize preclinical evidence for altered interneuron function in both positive and negative changes in brain function following anesthetic exposure. Finally, we propose approaches to elucidate the roles of interneurons in anesthetic mechanisms and discuss the advanced methods that could address current questions.

## Interneuron Subtypes: Physiological Properties and Synaptic Targets

We chose to focus this review on cortical and hippocampal interneurons in part because we have the most advanced understanding of the fundamental inhibitory systems governing excitatory neuron output and network function in these brain regions. However, anesthesia is a composite state with several distinct behavioral endpoints (unconsciousness, amnesia, immobility, and analgesia) attributed to different brain regions. For example, loss of consciousness alone involves not only the cortex but also the thalamus, subcortical regions, and the brainstem. It is difficult to compare cortical and hippocampal interneuron systems with those in other brain regions critical for anesthesia in part because the basic neuroanatomy differs. However, interneurons do share common features and embryological origins, such that the overlapping pharmacological effects may be generalizable.

**Figure 1:**
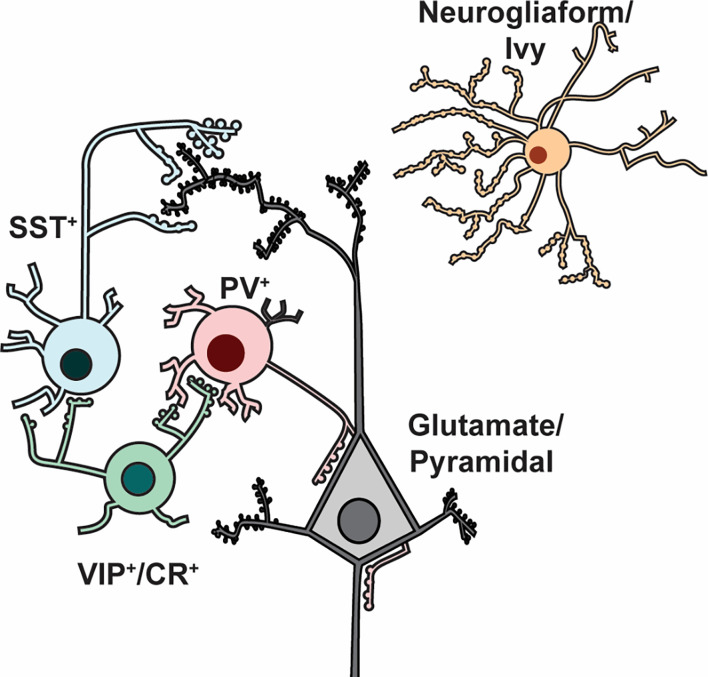
Major interneuron subtypes. Cartoon illustration of prominent interneuron subtypes of the cortex and hippocampus, showing their stereotypical arrangements with each other and with glutamatergic/pyramidal neurons. PV^+^ (parvalbumin-expressing) interneurons preferentially make axo-somatic and axo-axonic synapses onto glutamatergic pyramidal neurons, whereas SST^+^ interneurons target pyramidal neuron dendrites. VIP^+^/CR^+^ interneurons target PV^+^ and SST^+^ interneurons to disinhibit pyramidal neurons. Neurogliaform/ivy cell axons generally form a dense network of terminals that do not form classic synapses but instead release GABA volumetrically. This simplified diagram does not illustrate laminar distributions and morphology, which vary by region.

In the cerebral cortex and hippocampus GABAergic interneurons represent only ~10–20% of total neurons, however, they exert a disproportionately large impact on functional network outputs. By definition, inhibitory interneurons synthesize and release GABA by one of two glutamic acid decarboxylase isoforms (GAD65 and GAD67). However, many GABAergic interneurons also corelease various neuromodulators and neuropeptides that define them both immunocytochemically and functionally. Interneuron subpopulations include multiple subtypes (Figure 1) with characteristic phenotypes for modulation of excitability, action potential (AP) firing behavior, synaptic connectivity, and network-level functions. This broad functionality is driven largely by differential expression of protein effectors related to synaptic transmission (Paul et al., 2017).

Below we outline the defining characteristics of prominent cortical and hippocampal interneuron subtypes following conventional classification (Petilla Interneuron Nomenclature Group et al., 2008). Cortical and hippocampal interneuron subtypes can be discussed together because many of their characteristics and circuit roles are conserved; other review articles provide more detailed region-specific classifications (Pelkey et al., 2017; Lim et al., 2018). In general, distinct interneuron subtypes have characteristic neurophysiological properties and form stereotypical synaptic connections or microcircuits with excitatory neurons, which usually provide microcircuit output by innervating distinct subcellular targets (e.g., soma, dendrite, axon, bouton).

**Parvalbumin-expressing (PV^+^) interneurons** are the most extensively characterized interneuron subtype in terms of their cellular properties and circuit function. PV^+^ interneurons preferentially innervate excitatory neurons for fast phasic signaling and large-scale synchronized network activity (Hu and Jonas, 2014). Basket cells target the cell body and proximal dendrites, and chandelier cells target the axon initial segment. Functionally PV^+^ cells are typified by a “fast firing” (>50 Hz) response to depolarizing current injection without accommodation (diminished spike height during a train). In contrast, excitatory pyramidal neurons and most other interneurons have lower maximal firing rates and show spike accommodation with AP height rapidly declining during a sustained depolarization. PV^+^ cells have the highest maximal firing rate of hippocampal neurons (>300 Hz), critical for participation in high-frequency network activity. The AP waveform of PV^+^ interneurons is also unique compared to excitatory neurons and other interneurons, characterized by a narrow profile (short half-width) and a large, brief afterhyperpolarization due to high axonal expression of kinetically fast K^+^ channels (Hu et al., 2018). PV^+^ interneurons receive synaptic drive from local excitatory neurons and thalamocortical neurons and thus are sensitive to thalamocortical oscillations (Hafner et al., 2019). PV^+^ interneuron activity is further synchronized by extensive dendritic gap junction-mediated electrical coupling (Hu and Jonas, 2014).

PV^+^ synaptic terminals are specialized by a short delay and brief duration. In contrast to pyramidal neurons, which express multiple presynaptic Ca^2+^ channel subtypes with varying gating kinetics, PV^+^ terminals express only P/Q-type Ca^2+^ channels with fast gating kinetics. Synaptic Ca^2+^ channels and synaptic vesicles are arranged closer together for “tight” coupling with the Ca^2+^-sensitive synaptic vesicle exocytotic proteins (Hu et al., 2014). On a network level, PV^+^ interneurons control oscillatory rhythm generation and pacing and have been proposed to underlie gamma-band activity (20–80 Hz) in the cortical electroencephalogram (EEG; Cardin et al., 2009; Sohal et al., 2009). The relationship between PV^+^ interneurons and gamma frequency is being studied in relation to attention and memory, with implications for cognitive dysfunction in schizophrenia and Alzheimer’s disease (Lewis et al., 2012; Klein et al., 2016).

**Somatostatin-expressing (SST^+^) interneurons** are the primary source of axo-dendritic inhibitory synapses onto excitatory neurons in the hippocampus (Yavorska and Wehr, 2016).

Most cortical SST^+^ interneurons are further defined as Martinotti cells based on the morphological profile of the oriens lacunosum-moleculare (OLM) and a single prominent axon that projects superficially. Hippocampal SST^+^ interneurons, named OLM and hilar perforant path-associated (HIPP) neurons, are considered the functional homologs of cortical Martinotti cells and share a similar morphology^4^.

Martinotti SST^+^ neurons are non-fast firing, show spike adaptation, and have a maximum firing rate far lower than PV^+^ interneurons. The AP is wider due to differential voltage-gated Na^+^ and K^+^ channel expression and kinetics. SST^+^ interneurons have a higher intrinsic excitability than PV^+^ interneurons, with a lower AP threshold and high basal activity in the theta bandwidth (3–10 Hz). SST^+^ interneurons are primarily excited by pyramidal neurons and inhibited by VIP^+^ interneurons. VIP^+^/SST^+^ axo-dendritic synapses express GABA_A_ receptors containing the high-affinity α5 subunit consistent with strong inhibition (Magnin et al., 2019).

SST^+^ synapses target the distal dendrites of excitatory neurons and can selectively inhibit specific inputs, unlike PV^+^ interneurons that target excitatory cell bodies and soma post-integration to prevent firing (Urban-Ciecko and Barth, 2016). SST^+^ interneuron terminals release SST, a neuroactive peptide that exerts a variety of presynaptic and postsynaptic effects, mostly inhibitory (Liguz-Lecznar et al., 2016). SST is not co-released with GABA during normal synaptic vesicle exocytosis, rather it is released from dendritic and axonal dense-core vesicles following repetitive high-frequency stimulation. Because SST has antiepileptic activity, an endogenous homeostatic or protective role has been suggested (Tallent and Qiu, 2008). At the network, SST^+^ interneurons have a critical role in generating slow wave EEG activity associated with sleep (Funk et al., 2017).

## Interneuron-Selective/Disinhibitory Interneurons

About 20% of hippocampal and 15% of cortical interneurons express VIP and/or calretinin (CR) and selectively target other interneurons. This circuit role is thought to disinhibit local excitatory cells by creating holes in the “blanket of inhibition”, and/or modulate spatiotemporal patterns of inhibition and tune the excitatory cell input/output firing relationship (Karnani et al., 2014). VIP^+^/CR^+^ interneurons are part of the 5HT3_A_ receptor-expressing interneuron group discussed below (Lee et al., 2010).

VIP^+^ interneuron-selective interneurons show a variety of unique spiking behaviors during sustained depolarization including “irregular” behavior with single APs firing randomly; “bursting” behavior with three-five high-frequency APs discharged initially, followed by single APs at random intervals”; and “stuttering” behavior with clusters of spikes separated by silent periods of varying duration (Posluszny, 2019). VIP^+^ interneurons preferentially target SST^+^ interneurons and a fraction of PV^+^ interneurons (Turi et al., 2019).

Whether VIP is also released as a modulator from these interneurons is unknown. This is an interesting possibility since VIP applied directly to hippocampal CA1 and CA3 *in vitro* increases pyramidal cell excitability (Haas and Gahwiler, 1992). Related suprachiasmatic VIP^+^/GABAergic neurons release VIP for postsynaptic modulation of inhibitory postsynaptic currents and postsynaptic neuron firing rate(Itri and Colwell, 2003). About 15% of cortical and 30% of prefrontal VIP^+^ interneurons also co-release acetylcholine and participate in the regulation of attention (Obermayer et al., 2019).

*In vivo*, hippocampal VIP^+^ interneuron-selective interneurons are recruited at the peak of wake-state theta oscillations and are silent during ripples (Luo et al., 2020). In the prefrontal cortex, they are active during discrimination tasks. Guet-McCreight and colleagues reviewed recent studies into hippocampal and cortical VIP^+^ and CR^+^ interneuron-containing circuits, with special comparisons for distinct cortical regions with comparable actions (Guet-McCreight et al., 2020). Across these subregions, VIP/CR^+^ disinhibition commonly serves to control the integration of input to excitatory neurons and to control synaptic plasticity.

**Neurogliaform cells** of the cortex and hippocampus **and ivy cells** of the hippocampus mediate slow inhibition and share a unique morphology. An axonal “plexus” forms a high density of terminals without clear postsynaptic appositions (Armstrong et al., 2012). Instead of releasing GABA *via* classical synaptic exocytosis, these terminals release GABA in a volumetric “cloud” onto local processes that generates long-lasting inhibitory postsynaptic currents. Conceptually this volumetric release mode lies between phasic and tonic transmission (Olah et al., 2009; Overstreet-Wadiche and McBain, 2015). They also express a unique late-spiking AP phenotype that allows “slow integration” of input prior to firing. Spikes are non-accommodating and speed up during depolarization. Repetitive activity can elicit a late-spiking variation called barrage firing, where hundreds of APs occur at ~20–130 Hz for up to several seconds to minutes. This has been proposed to operate as an “excitability brake” (Sheffield et al., 2013; Overstreet-Wadiche and McBain, 2015).

Ivy cells and many neurogliaform cells also express neuronal nitric oxide synthase (nNOS) which creates the gaseous transmitter nitric oxide (NO) that has both presynaptic and postsynaptic actions (Hardingham et al., 2013). nNOS activity and NO signaling have been investigated as targets for volatile anesthetics, but roles remain unresolved. Volatile anesthetics alter NO production in cultured cerebellar neurons (Rengasamy et al., 1997; Loeb et al., 1998; Sjakste et al., 1999), however, NO loss of function animal models have shown both increased or decreased anesthetic sensitivity (Engelhardt et al., 2006; Nagasaka et al., 2017). Future investigations into these signaling pathways are needed to delineate the exact preclinical mechanisms and their contributions to the behavioral and clinical effects of anesthetics.

## Interneurons in Oscillations: Implications for The Macroscopic Rhythms of Anesthesia

The ensemble coordinated activity of distinct interneurons is thought to be critical for controlling global network activity and cognitive function (Buzsaki and Chrobak, 1995). This is supported by observations that interneuron subtypes preferentially fire at specific phases of oscillatory rhythms (Klausberger and Somogyi, 2008), and that interneuron subtype-specific perturbations alter oscillatory rhythms and behavior. How distinct interneurons shape the oscillation patterns that characterize induction and emergence from general anesthesia remains unclear mechanistically.

Modeling studies of patient forebrain EEG data collected intraoperatively usually represent synaptic connections as either excitatory or inhibitory. As a notable exception, Hashemi and colleagues found that tonic inhibition potentiated *via* extrasynaptic GABA_A_ receptors in interneurons could model power surges in δ and α frequency ranges characteristic of propofol anesthesia (Hashemi et al., 2014). Tonic inhibition correlates with the expression of GABA_A_ α5-containing receptors in SST^+^ interneurons and GABA_A_ δ-containing receptors in PV^+^ and neurogliaform/ivy interneurons which are very anesthetic sensitive (described below). In the basal forebrain associated with overlapping sleep and anesthesia circuits, selective activation of SST^+^ but not of PV^+^ interneurons results in increased sensitivity to isoflurane anesthesia and propofol anesthesia concomitant with potentiation of several EEG hallmarks of deep anesthesia including increased δ frequency power (Cai et al., 2021). How anesthetic potentiation of tonic inhibition in these cell types translates to cellular and circuit changes is not straightforward, as increased tonic inhibition has a cell-type-specific effect on neurophysiological parameters including gain control (Bryson et al., 2020).

Cell-type-based modeling can provide key insights into understanding network oscillatory behavior (Skinner, 2012; Keeley et al., 2017). Thus modeling studies of anesthetized human EEG data could be facilitated by incorporating elements that account for interneuron neurophysiological and pharmacological diversity.

## Interneuron Molecular Targets for Anesthesia

**Figure 2:**
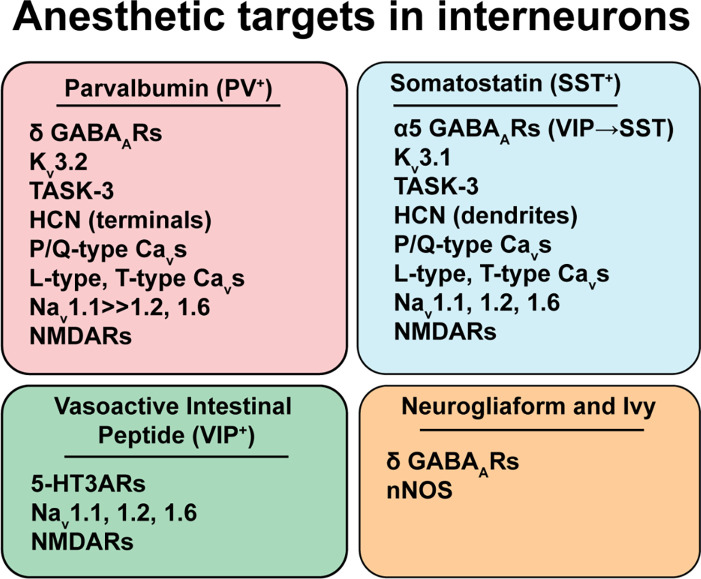
Anesthetic targets in interneurons. Highlighted anesthetic targets that are differentially expressed amongst the prominent interneuron subtypes from Figure 1. Anesthetic effects on specific targets and cells are complex and anesthetic agent-specific, so they are not shown in the figure. Please see the text for a discussion of the direction, potency, and implications of specific anesthetic effects. Abbreviations: GABA_ARs_, γ-aminobutyric acid type A receptors; K_v_3.1/K_v_3.2, voltage-gated potassium channels; TASK-3, TWIK-related acid-sensitive potassium channel subtype 3; HCN, Hyperpolarization-activated cyclic nucleotide-gated channels; Na_v_, voltage-gated sodium channels, with the Na_v_1.1 subtype being more abundant in PV^+^ interneurons; NMDAR_s_, N-methyl-D-aspartate receptors; 5-HT3_A_R, 5-hydroxytryptamine type 3A receptors; nNOS, neuronal nitric oxide synthase.

There are a number of identified anesthetic targets that are differentially expressed between interneuron subtypes. We highlight some examples with potential consequences for interneuron cellular and synaptic function (Figure 2). This is not meant to be an exhaustive review of known protein targets of volatile anesthetics, which have been reviewed recently (Hemmings et al., 2019). Rather, we focus on targets for which there are potential cell-type-specific effects such as evidence of differential expression between interneuron subtypes.

**K^+^ channels** control intrinsic excitability and firing patterns by hyperpolarizing the membrane potential at rest and repolarizing the membrane potential following APs. Several superfamily members are sensitive to anesthetics (Li et al., 2018; Riegelhaupt et al., 2018). For interneurons, K^+^ channel subtype and splice form expression are highly diverse, and several are associated with subtype-specific electrical characteristics (Casale et al., 2015; Pelkey et al., 2017). Voltage-gated K^+^ channels (K_v_) shape the AP waveform. K_v_3 channels are activated by depolarized membrane potential during the AP and mediate rapid repolarization, enabling hippocampal PV^+^ and SST^+^-OLM interneurons to fire at a higher frequency than pyramidal neurons (Rudy and McBain, 2001). Fast-firing PV^+^ interneurons utilize K_v_3.2 whereas non-fast firing SST^+^-OLM neurons utilize K_v_3.1 which has slightly slower deactivation kinetics (Pelkey et al., 2017). Although the sensitivity of K_v_3.1 to anesthetics has not been reported, other isoforms show interesting differences in pharmacology. Heterologously expressed K_v_3.2 is inhibited by volatile anesthetics yet activated by propofol, whereas K_v_3.4 is unaffected by either (Bhattacharji et al., 2010; Barber et al., 2011).

**Two pore domain K^+^ (K2P) channels**, including the Twik-related acid-sensitive K^+^ channel (TASK) subtype, mediate background leak conductance. Many forms of K2P are implicated in anesthesia and natural sleep cycles (Steinberg et al., 2015). Interneurons have relatively more TASK-3 and less TASK-1 compared to pyramidal neurons which express both. Heterologously expressed TASK-3 channels show greater potentiation by halothane than TASK-1 (Sirois et al., 2000). TASK-3 and TASK-1 knockout mice are both less sensitive to volatile anesthetics, have aberrant sleep/wake transition patterns, and their cortical EEG lacks theta oscillation (4–9 Hz; Pang et al., 2009). Because PV^+^ and SST^+^ interneurons are critical to theta oscillations and show strong immunoreactivity for TASK-3, this target may be a critical substrate for anesthetic effects on theta rhythms (Taverna et al., 2005).

**Hyperpolarization-activated cation channels (HCN)** participate in subthreshold excitability and rhythmicity; HCN inhibition by general anesthetics contributes to loss of consciousness and amnesia (Ying et al., 2006; Zhou et al., 2015). HCN channels are ubiquitously expressed among excitatory and inhibitory neurons, but different subcellular localization may impact anesthetic effects. Hippocampal excitatory neurons and probably SST^+^-OLM neurons express HCN channels mostly in the soma and dendrites (Zhou et al., 2015). In contrast, PV^+^ interneurons express HCN channels exclusively in axons and terminals which contribute to their high maximal AP frequency, so their fast-firing output may be anesthetic sensitive (Roth and Hu, 2020).

**Voltage-gated Na^+^ channels (Na_v_)** are inhibited by volatile anesthetics (with depressed peak current and enhanced inactivation) resulting in AP depression and reduced presynaptic excitability. The CNS Na_v_ channels have distinct anesthetic sensitivities and cellular expression patterns. Na_v_1.1 is enriched in interneurons (especially PV^+^) and is the least sensitive to volatile anesthetics, whereas Na_v_1.2 is more abundant in SST^+^ interneurons and is more sensitive to volatile anesthetics (Zhou et al., 2019). In addition to the voltage-gated channels associated with AP upstroke, Na^+^ leak channels (Ou et al., 2020; Yang et al., 2020) and persistently active channels (Zhao et al., 2019) are anesthetic-sensitive and contribute to anesthetic actions.

Commensurate with a critical role in PV^+^ interneuron AP kinetics, Na_v_1.1 in PV^+^ interneurons is necessary for normal oscillations at the network level, particularly gamma band activity, and for cognitive function. Na_v_1.1 expression is decreased in an Alzheimer’s disease model, and their PV^+^ interneurons show abnormal cellular and synaptic physiology. Genetic rescue of Na_v_1.1 ameliorated PV^+^ dysfunction, epileptiform discharges, and restored deficits in gamma band activity and cognitive function in this Alzheimer’s disease model (Verret et al., 2012).

The **ionotropic serotonin receptor (5-HT3_A_R)** is expressed in a large heterogeneous group of interneurons (~30% in the cortex, including VIP^+^/CR^+^ disinhibitory interneurons) that can be depolarized by 5-HT3_A_R agonists (Lee et al., 2010). Anesthetic effects on 5-HT3_A_Rs have not been extensively investigated as fundamental for hypnosis, likely because blockade does not alter anesthetic potency in rats (Rampil et al., 2001), and the consequence of 5-HT3_A_R modulation on interneuron activity is unknown. Heterologously expressed 5-HT3_A_R is allosterically potentiated by volatile anesthetics (Solt et al., 2005; Stevens et al., 2005).

Developmentally, 5HT/5HT3aR signaling is critical for several prenatal and postnatal mechanisms of interneuron circuit formation including cell migration (Vitalis et al., 2007; Murthy et al., 2014).

**Voltage-gated Ca^2+^ channel (Ca_v_)** subtypes differ in biophysical parameters, cellular expression profiles, and roles for synaptic plasticity (Vinet and Sik, 2006; Yamamoto and Kobayashi, 2018). Presynaptic Ca_v_2.1 and Ca_v_2.2 (producing P/Q-type or N-type currents, respectively) mediated Ca^2+^ entry controls synaptic vesicle exocytosis and neurotransmitter release. Excitatory synapses express a mixture of P/Q and N-type channels to mediate synchronous glutamate release (Dolphin and Lee, 2020), however, some interneurons show preferential expression of distinct forms coupled to GABA release. Hippocampal PV^+^ interneuron axosomatic terminals utilize fast P/Q-type channels for synchronous transmission. In contrast, axosomatic terminals from another type of interneuron expressing cholecystokinin (CCK^+^) utilize slower N-type channels and mediate asynchronous transmission (Hefft and Jonas, 2005; Zaitsev et al., 2007).

The question of how anesthetics affect P/Q- *vs*. N-type channel function is pertinent to understanding how distinct forms of inhibition are affected by anesthetics. Multiple studies demonstrate Ca_v_ inhibition by anesthetics, but relative potencies vary with the heterologous expression system, brain region, and cell-type (reviewed by Orestes and Todorovic (2010)).

Voltage-gated Ca^2+^ channels also influence Ca^2+^-gated second messenger signaling cascades for activity-dependent plasticity. **Ca_v_1.2/1.3 (L-type)** channel expression is highest in PV^+^ and SST^+^ interneurons. In PV^+^ interneurons, and L-type Ca^2+^ channels participate in a novel cell-specific pathway for CREB phosphorylation and activity-dependent transcriptional regulation (Cohen et al., 2016). PV^+^ interneuron development also involves an L-type Ca^2+^ channel-mediated transcriptional pathway (Jiang and Swann, 2005). Volatile anesthetics inhibit L-type channel conductance and G protein-coupled receptor signaling (Kamatchi et al., 2001; Fanchaouy et al., 2013). Thus L-type channel-dependent plasticity may be a target for changes in PV^+^ interneuron function following anesthetic exposure (see below). Ca_**v**_3 (T-type) channels have also been associated with distinct forms of plasticity between hippocampal PV^+^ and SST^+^ synapses onto pyramidal neurons (Udakis et al., 2020).

**GABA_A_ receptors** are well known as the major anesthetic target for postsynaptic facilitation of phasic and tonic inhibitory GABAergic signaling. Interneuron subtype-specific synapses differ in GABA_A_ receptor subunit expression (Sperk et al., 1997; Contreras et al., 2019), which determines receptor function and pharmacology (Pirker et al., 2000; Bonin and Orser, 2008).

Interneurons differentially express the high GABA affinity α4, α5, and δ subunits often associated with extrasynaptic receptors, which detect micromolar GABA from synaptic spill-over and/or volumetric release to mediate persistent or tonic inhibition (Ferando and Mody, 2014). Anesthetic modulation of tonic conductance dampens excitability by reducing firing frequency and hyperpolarizing the resting membrane potential. PV^+^ interneurons and neurogliaform cells are enriched in δ subunits and show a prominent tonic GABA current sensitive to δ specific ligands like neurosteroids (Ferando and Mody, 2015). This input is critical for normal network function, as PV^+^ interneuron-selective δ subunit deletion impairs CA3 gamma oscillation frequency (Mann and Mody, 2010). Cortical neurogliaform neuron expression of δ tonic conductance may act as a feedback mechanism for volumetric GABA release (Olah et al., 2009).

In contrast, hippocampal SST^+^ interneuron tonic inhibition is weak and insensitive to δ selective ligands (Vardya et al., 2008). Instead, SST^+^ neurons receive inhibition from VIP-expressing interneurons *via* dendritic synapses onto postsynaptic α5 subunit-containing receptors, which are high-affinity and normally found extrasynaptically (Magnin et al., 2019). GABA_A_ receptors containing α5 subunits are very sensitive to low concentrations of anesthetics (Caraiscos et al., 2004), and are upregulated by inflammation and anesthetic exposure, which is a possible mechanism for persistent cognitive impairment (Wang et al., 2012; Zurek et al., 2014). If α5 subunit-containing receptors at the SST^+^/VIP^+^ synapse are also upregulated, downstream SST^+^/excitatory synapses would be inhibited by anesthetic potentiation of α5-containing GABA_A_ receptors.

**NMDA-type glutamate receptors** are inhibited by volatile and dissociative anesthetics with varying potencies. Interneuron subtypes vary in their N-methyl-D-aspartate (NMDA) receptor subunit expression, which alters the kinetic properties of Ca^2+^ influx (Akgul and McBain, 2016). NMDA receptor signaling is important for PV^+^ interneuron function, and inhibition is associated with a variety of network changes including altered EEG gamma rhythm. NMDA receptor signaling in interneurons, especially the PV^+^ subtype, appears to be critical to ketamine’s rapid antidepressant effects. Subanesthetic doses are hypothesized to antagonize PV^+^ interneuron NMDA receptors selectively while sparing those at the pyramidal-pyramidal neuron synapse, thus reducing PV^+^ interneuron activity and disinhibiting pyramidal neurons (Homayoun and Moghaddam, 2007). This selective action at glutamatergic synapses has been attributed to the fast-firing phenotype of PV^+^ interneurons leading to a more depolarized resting membrane potential and greater relief of basal Mg^2+^ pore block, facilitating NMDA receptor-mediated excitation (Cohen et al., 2015). However, evidence suggests that other interneurons including the SST^+^ subtype may also participate in the antidepressant actions of ketamine (see below). NMDA receptor subunits also control interneuron development in a subtype-specific manner, so distinct anesthetic sensitivities could result in different consequences from exposure during the critical period of neurodevelopment (De Marco Garcia et al., 2015).

## Acute Interneuron Subtype-Specific Anesthetic Effects

Inhibitory interneurons exert subtype-specific control of excitatory neuron firing patterns at the microcircuit level, and at a microcircuit network level reflected in oscillatory rhythms. Subtle disturbances in cellular function have a cascading impact on network function, as distinct effects on interneuron excitability distort normal action potential output, and alter their contributions to macroscopic rhythms. These large-scale alterations of the excitation/inhibition balance may be a basis for altered neural function observed in association with anesthetic exposure (see below). Given the complexity of neuronal circuits and the delicate balance between excitation and inhibition, transmitter-specific effects can produce opposite neurophysiologic results. For example, enhanced inhibitory input onto inhibitory interneurons can lead to disinhibition, or indirect excitation by removal of an inhibitory input (Karnani et al., 2014). Despite these important functional implications, interneuron subtype-selective anesthetic actions have been poorly characterized. The following sections highlight representative examples of known acute anesthetic effects and their implications.

**Fast-firing/parvalbumin interneurons** Specific anesthetic effects on both intrinsic and synaptic properties have been observed in interneurons with a fast-firing phenotype consistent with PV^+^ interneurons. In the insular cortex, fast-spiking interneurons are less sensitive to propofol depression of membrane excitability compared to other interneurons (regular and late-firing), and especially compared to pyramidal neurons (Kaneko et al., 2016). This is due to differential changes in tonic inhibition and its potentiation by propofol. Fast-spiking interneuron synapses onto pyramidal neurons also show greater facilitation by propofol compared to those from non-fast-spiking interneurons (Koyanagi et al., 2014). Together these observations suggest distinct anesthetic actions of fast-firing interneurons and axo-somatic inhibition of pyramidal neurons. However, at subanesthetic concentrations of isoflurane hippocampal fast-spiking interneuron firing rate is decreased concomitant with an increase in pyramidal neuron firing rate. This suggests that distinct drug effects on a given cell type can emerge at different stages of anesthesia (Zhao et al., 2021).

**SST^+^ neuron** activity *in vivo* is very sensitive to isoflurane and urethane (Urban-Ciecko and Barth, 2016). Cortical SST^+^ interneuron visual field tuning is markedly more sensitive to anesthetics compared to pyramidal neurons and PV^+^ interneurons, with profound anesthetic depression of visual stimulus-evoked spiking specific to SST^+^ but not PV^+^ interneurons (Adesnik et al., 2012). SST^+^ interneuron excitability is also sensitive to ketamine. Recordings *in vivo* show that ketamine depression of SST^+^ interneuron activity allows pyramidal neuron disinhibition, with even subanesthetic doses enabling increased pyramidal neuron dendritic Ca^2+^ influx (Ali et al., 2020). SST^+^ interneurons overlap with regular-firing insular interneurons that show greater sensitivity to propofol inhibition of intrinsic excitability compared to fast-firing interneurons (Kaneko et al., 2016).

*In vivo* recording of PV^+^ and SST^+^ interneurons during up- and down-state transitions during urethane anesthesia show different activity profiles in terms of both frequency and phase relation to slow local field potential changes (Zucca et al., 2017). More than just reflecting different responses to anesthesia, these neurons may have different functional roles in cortical state transitions. Optogenetic manipulation of PV^+^ interneuron activity has a greater effect on state transitions, and both endogenous and induced PV^+^ firing is associated with delayed transition from down- to up-state.

The role of SST^+^ interneurons in endogenous sleep circuits raises the possibility that they also operate in overlapping anesthesia/sleep circuits (Funk et al., 2017; Moody et al., 2021). In the basal forebrain, select SST^+^ interneuron activation potentiates propofol and isoflurane hypnosis, with a comparably smaller contribution from PV^+^ interneurons (Cai et al., 2021).

**Irregular-spiking interneurons** at the border of hippocampal stratum lacunosum-moleculare and stratum radiatum exhibit diverse pharmacological properties in rat (Nishikawa and MacIver, 2000). In neurons expressing I_h_ (HCN-mediated) currents, ~60% show I_h_ potentiation by halothane with no effect in the rest. In most interneurons, halothane decreases spontaneous firing rate, but for ~8% of interneurons, halothane depolarizes the resting membrane potential and increases spontaneous firing. Halothane, isoflurane, and sevoflurane all increase spontaneous inhibitory postsynaptic current frequency, consistent with increased presynaptic release probability at unidentified local interneuron-interneuron connections (Nishikawa and MacIver, 2001).

**VIP^+^ interneuron** activity disinhibits excitatory neurons *via* inhibition of PV^+^ and SST^+^ interneurons, which can be observed with isoflurane anesthesia. In the visual cortex, VIP^+^ interneuron responses to visual stimuli are similar in awake or anesthetized animals, in contrast to SST^+^ interneurons which show a dramatically different response to the same stimulus depending on the awake or anesthetized state (Jackson et al., 2016). This response profile suggests that, unlike SST^+^ interneurons, VIP^+^ interneurons and their network actions are resistant to anesthesia, although further investigations are needed to delineate whether this apparent anesthetic insensitivity reflects intrinsic cellular pharmacology. As in awake animals, selective optogenetic activation or chemogenetic inhibition of VIP^+^ interneurons can increase or suppress local network activity, respectively under anesthesia (Karnani et al., 2016). VIP^+^ interneuron activity correlates with spontaneous changes in local network activity with isoflurane anesthesia and is an interesting candidate for the circuit basis of fluctuations in activity during stable anesthesia such as burst suppression.

A recent study analyzed interneuron-specific intracellular Ca^2+^ changes observed immediately after the loss of consciousness (Guo et al., 2021). VIP^+^ interneurons showed the highest activity compared to PV^+^ and SST^+^ interneurons, even higher than prior to isoflurane exposure. Whether this simply reflects VIP^+^ interneuron disinhibition due to global activity changes or a more active role in generating hypnosis remains to be tested functionally.

## Evidence That Anesthetics Promote Interneuron Dysfunction

**Figure 3:**
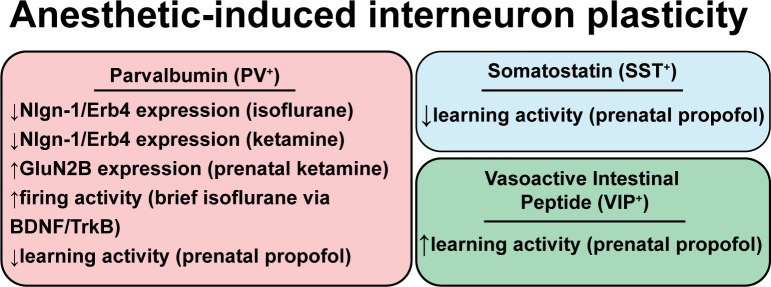
Anesthetic-induced interneuron plasticity. Review of evidence for persistent changes in interneuron function following anesthetic exposure. Abbreviations: Nlgn1/Erb4, signaling between neuroligin-1 and its receptor ErbB4; GluN2b, N-methyl D-aspartate receptor subunit 2B; BDNF, brain-derived neurotrophic factor; TrkB, tropomyosin receptor kinase B.

Isoflurane, ketamine, and urethane anesthesia all elicit short-term changes in evoked inhibitory responses, suggesting plasticity of inhibitory synapses, as normal interneuron-excitatory neuron connections are altered during anesthesia (Taub et al., 2013). There is also evidence for long-term changes that persistently alter cognitive function (Vutskits and Xie, 2016). Figure 3 highlights examples of interneuron plasticity or altered function associated with exposure to anesthetics.

Volatile anesthetics have been implicated in persistent neurocognitive dysfunction following emergence from anesthesia (Evered et al., 2018), especially in aged and sick individuals, with several preclinical animal models under active study (Eckenhoff et al., 2020). Interneuron cellular and synaptic dysfunction are implicated in neurocognitive dysfunction, including age-related cognitive decline (Rozycka and Liguz-Lecznar, 2017), raising the possibility that these mechanisms are involved. Upregulated GABAergic signaling *via* α5 subunit-containing receptors (Orser and Wang, 2019) implies changes in excitatory/inhibitory balance, consistent with the clinical phenotypes of hypoactivity and impaired working memory. Hippocampal neuroligin 1-Erb4 signaling, which is mostly localized to PV^+^ interneurons and mediates synaptogenesis and synaptic plasticity, is disrupted in aged mice following isoflurane exposure, and its rescue ameliorates the working memory impairment observed days after anesthesia (Li et al., 2014). Another study in aged mice suggests that PV^+^ interneuron death from oxidative stress precipitates cognitive impairment following laparoscopy with isoflurane anesthesia, a phenomenon that probably involves surgery-associated neuroinflammation (Qiu et al., 2016).

## Antidepressant Effects of Anesthetics, and A Potential Role of Interneuron Plasticity

The intravenous anesthetic ketamine has gained attention for eliciting rapid, persistent antidepressant effects from single subanesthetic doses in clinical studies (Berman et al., 2000). PV^+^ and SST^+^ interneurons in the prefrontal cortex have been identified as critical mechanistic initiators. Ketamine inhibition of GluN2B-NMDA receptor signaling in these interneurons is essential for its antidepressant-like effects in rodents (Gerhard et al., 2020). Inhibition of these interneurons and resulting disinhibition of pyramidal neurons results in a surge of glutamate and activation of plasticity mechanisms including increased BDNF release, TrkB, mTOR, and GSK3β signaling, and synaptic protein upregulation (Luscher et al., 2020). Both inhibitory and excitatory synaptic plasticity have been observed in animal models, as reviewed elsewhere(Luscher et al., 2020). For example, neuregulin 1-Erb4 signaling downregulation in PV^+^ interneurons contributes to the antidepressant effects of ketamine (Wang et al., 2014). In support of the idea that ketamine disrupts neuregulin 1-ErB signaling in PV^+^ interneurons to induce inhibitory synaptic plasticity, subanesthetic ketamine can reactivate adult visual plasticity in an ocular dominance model *via* PV^+^ interneuron neuroligin 1 downregulation and loss of excitatory input (Grieco et al., 2020).

Although less well understood, some clinical data suggest that isoflurane anesthesia can also elicit antidepressant effects and BDNF-mediated neural plasticity in interneuron function. Brief deep isoflurane anesthesia has antidepressant effects comparable to electroconvulsive therapy in human studies (Langer et al., 1985; Engelhardt et al., 1993; Weeks et al., 2013), although inconsistent findings have been reported (Greenberg et al., 1987; Garcia-Toro et al., 2001). Burst suppression has been proposed to replicate the effects of electroconvulsive therapy. In support of an antidepressant effect of isoflurane, research with rodent models found a reversal of depression biomarkers after isoflurane anesthesia with EEG-confirmed burst suppression (Brown et al., 2018). A study investigating antidepressant effects of burst-suppressing isoflurane anesthesia in a rodent model found that some elements of the ketamine antidepressant molecular pathway were replicated, including prefrontal TrkB and GSK3β activation (Antila et al., 2017). There was evidence for interneuron plasticity after anesthesia with a TrkB-dependent increase in PV^+^ interneuron activity, and robust immediate-early gene FosB staining in PV^+^ but not SST^+^ interneurons (Antila et al., 2017). Given that this increased PV^+^ interneuron excitability deviates from the ketamine model of pyramidal cell disinhibition, it will be interesting to see how these molecular events ultimately compare and correlate with antidepressant effects between specific anesthetics.

## Developmental Plasticity

There is also mounting evidence that anesthetics impact developmental plasticity in a cell-type specific manner, raising the possibility that interneuron dysfunction contributes to the long-term cognitive effects of neonatal or prenatal anesthesia. Studies characterizing interneuron development using pan-GABAergic labels show impaired interneuron morphology and function following ketamine exposure (Vutskits et al., 2006, 2007; Aligny et al., 2014). Subtype-specific genetic labeling and functional analysis reveal interesting complexities. Adult interneuron density is sensitive to midazolam anesthesia in a cell-type and developmental-stage specific manner, probably because interneurons undergo subtype differentiation asynchronously (Osterop et al., 2015). A recent study showed that neonatal propofol exposure differentially altered adult interneuron activity during motor learning, with SST^+^ and PV^+^ interneurons being hypoactive and VIP^+^ interneurons being hyperactive compared to vehicle-treated mice, suggesting abnormal synaptic integration and circuit formation (Zhou et al., 2021). Adult PV^+^ interneurons in ketamine treated mice show abnormal synaptic response properties due to altered NMDA receptor subunit expression, specifically an increase in sEPSC frequency and upregulation in NR2B/GluN2B subunits expression (Jeevakumar and Kroener, 2016).

## The Path Forward: Unanswered Questions and Approaches

Cortical neuron subtype-specific roles have been identified in slow oscillation up- and down-state transitions during sleep and isoflurane (Zucca et al., 2017), and ketamine (Kuki et al., 2015) anesthesia. Are these effects causal in generating loss of consciousness or amnesia? The possibility of circuit-specific anesthetic effects also raises questions for experimental neuroscience since anesthesia is routinely used during *in vivo* electrophysiological recordings that form the basis for most of what is known about *in vivo* neurophysiology. The possibility that anesthesia could mask or distort cellular properties is well known, for example, isoflurane and pentobarbital produce profound differences in auditory cortical neuron response properties (Cheung et al., 2001). Are there also neuron subtype-specific anesthetic actions or sensitivities that affect neurophysiology in anesthetized animals and thereby complicate data interpretation? How do interneuron subtype-specific sensitivities to anesthetics contribute to network dynamics, oscillatory behavior, and the intraoperative EEG?

Interneuron research has been revolutionized by the use of transgenic mouse models to identify or manipulate genetically defined neurons (Taniguchi et al., 2011; Daigle et al., 2018). Genetic labeling by fluorescent protein biosensor expression is widely used for neuron subtype identification in targeted recordings or anatomical studies. *In vivo* strategies have been especially powerful to delineate subtype-specific circuit and network function. Optogenetic and chemogenetic methods have been useful in isolating subcortical nuclei critical for hypnosis and arousal and could be applied to interrogate specific neocortical and hippocampal interneuron subtype roles.

As an alternative to genetic labels, interneurons can also be identified by AP waveform and spike timing in relation to known subtype-specific activity during theta and sharp-wave ripples (Kuang et al., 2010). This approach was used to identify diverse interneuron firing patterns under ketamine anesthesia, ranging from no change to induction of rhythmic activity not observed during slow-wave sleep or awake states.

**Transcriptomics** has illuminated how different programs of gene expression drive phenotypic specialization. A recent meta-analysis suggests that genes related to synaptic transmission are the greatest source of variation associated with neuronal subtype (Paul et al., 2017; Huang and Paul, 2019). Data also suggest that synapses show subtype specialization of presynaptic protein expression (Contreras et al., 2019).

***Ex vivo* brain slices** provide an excellent system in which to investigate anesthetic action on interneuron subtype-specific circuit effects because they allow targeted intracellular recordings and pharmacological accessibility while largely maintaining synaptic connectivity, even generating intrinsic oscillatory rhythms similar to those observed in anesthetized patients (Voss et al., 2019). Brain slices can be maintained for weeks in organotypic culture to investigate long-term changes associated with anesthetic exposure models (Drexler et al., 2010), which provide a valuable model for analyzing long-term anesthetic effects on synaptic plasticity.

This method has enabled site-specific pharmacologic investigations of synaptic modulation (Joksovic et al., 2015; Rodgers et al., 2015), as well as optogenetic (Murphy et al., 2020) and chemogenetic (Jiang-Xie et al., 2019) studies of anesthetic action, all of which hold promise to help delineate cell-type specific and circuit-level pharmacology. However, this system has its own limitations and technical variations that must be considered when comparing data (Humpel, 2015).

## Neuroplasticity

Although general anesthetics are clinically invaluable for their reversible short-term effects on consciousness, evidence is accumulating that they also have the potential to induce neuronal plasticity and long-term changes in cognitive function (Vutskits, 2012; Platholi and Hemmings, 2021). These outcomes appear steeply dependent not just on drug regime but also on human and animal model properties, e.g., clinical condition, age, surgical treatment, etc. Further investigations into the roles of interneuron subtypes in long-term adverse effects of anesthetics will have to account for immune-mediated changes in interneuron function, for example, PV^+^ interneuron activity increases with neuroinflammation (Feng et al., 2021).

Most mechanistic studies to date specifically investigating interneurons have focused on the PV^+^ subtype. Since different interneurons undergo distinct forms of plasticity, further studies will have to account for interneuron heterogeneity. Given the functional and pharmacological diversity of interneuron responses to acute anesthetic exposure, it will be important to determine whether long-lasting anesthetic effects are also cell-type specific.

## Generalizability

In this review, we focused on interneurons of the cortex and hippocampus, but an important question for anesthetic mechanisms is how much of this pharmacology extends to interneurons in other regions that contribute to anesthesia, such as the thalamus, basal forebrain, brainstem, spinal cord, etc? It is far too early to say, especially as investigations into interneuron diversity and characterization of anesthetic-sensitive molecular target expression in these regions are still ongoing. Although the fundamental architecture of these regions is wholly different making a comparison difficult if not impossible, a few striking similarities (such as the presence of interneurons expressing PV^+^ in all these regions) do beg the question of whether any of these associations are recapitulated elsewhere in the brain. For example, there are PV^+^ interneurons in the thalamus, although these are not from the same interneuron progenitor pool as cortical and hippocampal PV+ interneurons (Jager et al., 2021). Basal forebrain interneuron diversity appears similar to cortex and hippocampus, with PV^+^, SST^+^, and nNOS^+^ interneurons. Basal forebrain PV^+^ interneurons are also a large subset with fast-firing with narrow AP waveforms, although intrinsic membrane properties including HCN current expression appear to differ from cortical and hippocampal PV^+^ interneurons (McKenna et al., 2013; Yang et al., 2017). For now, we cautiously suggest circuit roles need to be investigated in a region-specific manner, but that the genetic strategies used to identify interneuron subtype-specific function and pharmacology in the cortex and hippocampus can also be used in other regions. Given that cortical and hippocampal interneurons show great homology and share a common embryological origin of interneuron progenitors, it is possible that other regions with common interneuron precursors may also share adult functional and pharmacological properties relevant to anesthesia.

## Summary

Studies of interneuron cellular and circuit function are rapidly advancing neuroscience research. Recent technical advances have revealed important principles for how inhibitory and excitatory neuron ensembles collectively shape neuronal circuit activity and information transfer. However, much of the known anesthetic pharmacology at specific molecular targets has yet to be tested at the interneuronal cellular, microcircuit, or network levels, and predicted interactions will need to be experimentally tested to evaluate their ultimate contributions to the anesthetic behavioral endpoints Application of this knowledge and associated technical advances to the investigation of anesthetic mechanisms will contribute to the elucidation of many long-standing questions regarding the complex actions of general anesthetics on CNS circuit function while also illuminating interneuron subtype-specific neurophysiological function.

## Author Contributions

IS and HH both participated in the concept and design of the review, writing, editing, and final approval of the manuscript. All authors contributed to the article and approved the submitted version.

## Conflict of Interest

HH is the editor in chief of the British Journal of Anesthesia, is a consultant for Elsevier (Philadelphia, PA, USA), and receives research funding unrelated to this study from Instrumentation Laboratory/Werfen (Bedford, MA, USA). The remaining author declares that the research was conducted in the absence of any commercial or financial relationships that could be construed as a potential conflict of interest.

## Publisher’s Note

All claims expressed in this article are solely those of the authors and do not necessarily represent those of their affiliated organizations, or those of the publisher, the editors and the reviewers. Any product that may be evaluated in this article, or claim that may be made by its manufacturer, is not guaranteed or endorsed by the publisher.
